# Knowledge–Behavior Relationships and Technology Adoption Among Patients With Diabetes: A Mixed‐Methods Analysis of Smart Foot Care Technology

**DOI:** 10.1002/jfa2.70051

**Published:** 2025-05-10

**Authors:** Ting‐Ting Yeh, Jawl‐Shan Huang, Yun‐Chieh Chou

**Affiliations:** ^1^ Master Degree Program in Health and Long‐Term Care Industry College of Medicine Chang Gung University Taoyuan Taiwan; ^2^ Division of Endocrinology and Metabolism Chang Gung Memorial Hospital at Linkou Taoyuan Taiwan

**Keywords:** diabetic foot care, health knowledge, mixed‐methods research, self‐management behavior, smart healthcare technology, technology adoption, type 2 diabetes

## Abstract

**Background:**

Although recent systematic reviews indicate low adherence to foot care practices among patients with type 2 diabetes compared to other self‐management behaviors, smart healthcare technologies offer potential solutions for improving foot care management. The smart diabetic foot screening system represents an innovative approach to diabetic foot care. However, the factors influencing its adoption, particularly the relationship between knowledge, behavior, and technology acceptance, remain poorly understood.

**Methods:**

A mixed‐methods design was employed, integrating quantitative and qualitative data. Quantitative data were collected from 80 patients with type 2 diabetes using validated instruments: the foot care knowledge questionnaire, diabetic foot self‐management behavior scale, and the unified theory of acceptance and use of technology questionnaire. Pearson correlation and regression analyses examined relationships between knowledge, behavior, and technology adoption intention. In‐depth, semistructured interviews with 20 participants explored adoption factors. Thematic analysis was conducted on qualitative data.

**Results:**

Despite high levels of foot care knowledge (86.2% correct response rate), actual self‐management behaviors remained suboptimal, with a modest correlation between knowledge and behavior (*r* = 0.31 and *p* < 0.01). Regression analysis identified attitude and facilitating conditions as significant predictors of smart system adoption intention, explaining 57% of the variance. Qualitative analysis revealed three main themes: technology acceptance perceptions, implementation support system, and self‐management patterns, highlighting the complex interplay between knowledge, attitudes, and behavioral factors.

**Conclusions:**

This study reveals that despite improved knowledge levels compared to previous decades, the knowledge–behavior gap in diabetic foot care persists. The findings suggest that successful implementations of smart healthcare technologies require addressing both attitudinal factors and facilitating conditions, rather than focusing solely on knowledge enhancement. These insights contribute to understanding technology adoption in chronic disease self‐management and inform the development of more effective implementation strategies.

## 1 Introduction

Diabetes mellitus constitutes a significant global health burden, with the International Diabetes Federation reporting approximately 537 million affected adults in 2021 [1]. Projections indicate that this number will escalate to 643 million by 2030 and 783 million by 2045, with an estimated 240 million adults currently undiagnosed [1, 2]. Among the numerous complications associated with diabetes, diabetic foot ulcers (DFUs) emerge as a particularly concerning sequela, with patients facing a 34% lifetime risk of development [3]. DFUs are characterized by full‐thickness skin loss on the foot, resulting from neuropathic and/or vascular complications in patients with diabetes mellitus [4]. The clinical significance of DFUs is underscored by their high prevalence and severe outcomes: approximately one‐third of diabetic patients will develop a DFU during their lifetime, with 75%–85% of cases progressing to infection and gangrene, potentially necessitating lower limb amputation [5]. Global statistics indicate that diabetes‐related amputations occur every 30 s [6], with diabetic individuals facing up to 20 times greater risk of amputation compared to nondiabetic populations [7].

The impact of DFUs extends beyond physical manifestations to significantly affect psychosocial well‐being. Research demonstrates that DFUs contribute to diminished independence and psychological distress [8]. Notably, one‐third of patients experiencing their first DFU develop clinical depression, which correlates with increased mortality rates [9]. Given the projected growth in global diabetes prevalence, the incidence of DFUs and subsequent lower limb amputations is expected to rise proportionally. This epidemiological trend emphasizes the critical need for high‐quality efficient screening tools for early detection and intervention [1, 10]. Despite significant advances in diabetes management and preventive foot care protocols, DFUs continue to present substantial clinical challenges [3, 11, 12]. Several critical barriers to effective prevention have been identified, including inadequate patient education regarding risk factors and preventive measures [13], limited accessibility to specialized podiatric care [14], suboptimal adherence to recommended foot care protocols [15], and financial constraints affecting preventive care access [14]. Healthcare access limitations, particularly in rural and underserved areas, further compound the challenges of implementing timely preventive measures [16, 17].

Recent technological innovations in DFU prevention and management have focused on patient‐centered care and self‐management strategies as documented in a comprehensive narrative review [18]. Emerging home‐monitoring technologies include intelligent smart socks [19, 20, 21], smart insoles [22, 23], and smart mats [24]. These devices enable real‐time monitoring of foot health parameters. However, despite these technological advancements, ulcer recurrence rates remain persistently elevated, with etiology appearing multifactorial, encompassing both biological mechanisms and behavioral factors [3]. The FootHow system is a prototype smart technology being developed in our research group for early detection and monitoring of DFU risk factors. The proposed system integrates advanced imaging capabilities with temperature assessment, designed to enable regular foot screening and monitoring in home settings. Once fully developed, this system aims to provide a user‐friendly platform for home‐based diabetic foot monitoring. Although the system is currently in its developmental phase, this study sought to understand potential users' perspectives and adoption factors to inform its further development.

The current study addressed two primary objectives: first, to examine the relationship between foot care knowledge and self‐management behavior in patients with diabetes and second, to identify the determinants of behavioral intention to adopt the FootHow system using a mixed‐methods approach. Through this comprehensive investigation, the study analyzed factors influencing technology adoption while exploring the connection between patients' understanding of foot care and their actual self‐management practices. This analysis provided crucial insights into the factors that facilitated or impeded patient adoption of smart DFU screening technology, thereby informing the development of more effective implementation strategies.

## 2 Methods

We employed an explanatory sequential mixed‐methods design [25, 26], combining quantitative and qualitative phases. The initial quantitative phase utilized four validated questionnaires to address primary research questions. Using G*Power v3.1 [27], we calculated a minimum sample size of 80 participants to detect a medium effect size (*f* ^2^ = 0.2) with 80% power (*α* ≤ 0.05 and *β* = 0.20) [27, 28]. The qualitative phase was subsequently designed to further interpret and elaborate on the quantitative findings [26]. The study protocol received approval from the Chang Gung Memorial Hospital Institutional Review Board (IRB: 202300512B0). The study population consisted of adult outpatients with diabetes mellitus from the endocrinology clinic at Chang Gung Memorial Hospital, Likou. Participants were identified and recruited through the outpatient center of the endocrinology clinic. To reach potential participants, recruitment posters were displayed in the clinic. Interested individuals were instructed to contact our research personnel directly to express their interest and learn more about the study. Inclusion criteria were adults ≥ 20 years old, diagnosed with diabetes for ≥ 1 year, clinically stable, able to communicate clearly, and willing to provide informed consent. Exclusion criteria included sensory impairments, psychiatric disorders, cognitive impairment, critical illness, minors, pregnant women, and individuals unable to read or write.

### 2.1 Phase 1: Quantitative Phase

The initial phase employed a population‐based cross‐sectional design to assess three key domains: diabetic foot self‐management behavior, foot care knowledge, and technology adoption intention. Three validated instruments were utilized: the diabetes foot self‐care behavior scale, foot care knowledge questionnaire, and the unified theory of acceptance and use of technology (UTAUT) questionnaire for innovative system adoption behavior assessment.

### 2.2 Diabetes Foot Self‐Care Behavior Scale

The study employed the diabetes foot self‐care behavior scale developed by Chin and Huang [29], comprising seven items across two subscales: execution and frequency. The four‐item execution subscale measures weekly foot self‐care practices using a 5‐point Likert scale (0–7 days), with an example item: “I (or my caregiver) can assist to check my feet.” The three‐item frequency subscale assesses overall foot self‐care behavior prevalence, also using a 5‐point Likert scale, exemplified by: “Before putting on your shoes, check whether there are any foreign objects such as small stones inside the shoes.” Previously validated in a Taiwanese population (Cronbach's *α* = 0.73) [29, 30], the scale demonstrated excellent internal consistency in this study (Cronbach's *α* = 0.91). Higher scores indicate more frequent and consistent diabetes foot self‐care behaviors.

### 2.3 Foot Care Knowledge Questionnaire

The questionnaire [31] is a 20‐item true/false instrument assessing diabetic foot care knowledge. The questionnaire evaluates patients' understanding of foot lesion etiology, examination procedures, problem identification, and daily care maintenance. With a maximum score of 20, higher scores reflect greater foot care knowledge. Previously validated in Taiwanese populations, the instrument demonstrated content validity through expert review and acceptable internal consistency (Cronbach's *α* = 0.75) [31].

### 2.4 Innovative System Adoption Behavior UTAUT Questionnaire

We utilized the UTAUT questionnaire [32], contextually modified to assess psychosocial determinants of behavioral intention toward the FootHow technology adoption for diabetic foot ulcer prevention. Participants received standardized multimodal information about the smart technology through written materials, verbal explanation, and video demonstration during the informed consent process, ensuring comprehensive understanding while maintaining the instrument's core construct validity.

The modified UTAUT instrument comprised eight domains: seven psychosocial constructs and one behavioral outcome measure, assessed through 28 items on a 5‐point Likert Scale (0 = strongly disagree to 5 = strongly agree). The validated constructs explored performance expectancy, effort expectancy, attitude, social influence, self‐efficacy, anxiety, and facilitating conditions. The behavioral intention evaluated participants' intention to adopt smart technology (Table 1). Composite domain scores were calculated by systematically aggregating corresponding item responses, maintaining theoretical integrity while enabling context‐specific assessment of technology acceptance in diabetic foot ulcer prevention [33, 34, 35].

**TABLE 1:** Psychosocial factors, definitions, and sample items from the modified UTAUT questionnaire for the FootHow system.

Psychosocial factor	Definition	Items	Example item
Performance expectancy	The extent to which an individual believes that using the system will improve their job performance	4	I believe FootHow would be useful for managing my foot health
Effort expectancy	The extent to which an individual perceives the system to be easy to understand and use	4	I expect FootHow's operation to be clear and easy to understand
Attitude	Individual's positive or negative feelings toward using the system	3	I think using FootHow is a good idea
Social influence	The degree to which an individual perceives that important people in their life believe they should use the system	3	People who influence my behavior think that I should use FootHow
Self‐efficacy	The extent to which an individual believes they possess the skills to adopt a system	3	I can use FootHow according to the instructions provided to me
Anxiety	Self‐reported level of anxiety or hesitation an individual experiences regarding the use of the system	4	I worry that if I press the wrong button, my data might not be recorded
Facilitating conditions	The degree to which an individual believes that organizational and technical infrastructure exists to support the use of the system	4	I have the necessary resources and knowledge to use FootHow
Behavioral intention	The extent to which an individual has consciously formulated intentions to engage in or refrain from performing a specified future behavior	3	I intend to adopt FootHow within the next year

*Note:* FootHow is a prototype smart technology being developed for early detection and monitoring of diabetic foot ulcer risk factors.

All questionnaires were administered in paper format through face‐to‐face interactions. For participants who could self‐complete (*n* = 42), the paper questionnaires were provided directly with instructions for completion. However, recognizing literacy challenges among some older participants (*n* = 38), trained research personnel verbally administered identical questionnaire items, reading each question aloud and documenting participants' verbal responses on the paper forms. This adaptive approach ensured consistent data collection although accommodating varying literacy levels and capabilities among our diverse sample. All questionnaire administrations occurred in private rooms at the diabetes clinic to maintain confidentiality.

### 2.5 Phase 2: Qualitative Phase

Participants expressing interest in the semistructured interviews voluntarily provided contact information at the questionnaire's conclusion. The study employed a qualitative descriptive research methodology [36] utilizing face‐to‐face interviews to explore in‐depth insights into smart diabetic foot screening technology adoption. Researchers conducted interviews using a structured protocol designed to probe questionnaire findings and uncover nuanced perspectives on technology integration. Targeted inquiries focused on identifying barriers and facilitators to incorporating smart foot screening technology into daily self‐management routines. Representative interview questions explored participants' perceived challenges and motivational factors in adopting such innovative healthcare technologies. The interviews were conducted by a trained researcher with a background in diabetes education and qualitative research methodology. This researcher was not involved in the participants' clinical care and was not previously known to them, which helped minimize potential response bias. The interviews followed a standardized protocol to ensure consistency while allowing for the exploration of individual perspectives.

Interviews were audio‐recorded and transcribed verbatim using Express Scribe (5.88, NCH Software, USA), with postinterview field notes documenting researcher reflections [37]. In cases of interruption, session summaries were documented and verified by participants, ensuring data trustworthiness [38]. Transcribed data were analyzed using NVivo for Mac (version 12) through systematic thematic analysis. Two independent researchers conducted coding using both a preliminary broad‐brush approach and a priori thematic categories derived from UTAUT domains [39]. Data not aligning with UTAUT domains underwent separate analysis for emergent themes [40]. Theme refinement occurred through comparative analysis and systematic deliberation among researchers until thematic saturation was achieved [41], ensuring comprehensive coverage of the study objectives. Although conflicts were minimal, any discrepancies that arose during coding were resolved through iterative discussions and consensus building among the research team, ensuring that the final themes accurately reflected the data.

## 3 Data Analysis

Quantitative analyses were performed using IBM SPSS Statistics (Version 25; IBM Corporation, NY), with statistical significance set at *p* < 0.05. Nominal data were reported as proportions and ratio data as mean and standard deviation. Internal consistency reliability of the UTAUT was evaluated using Cronbach's alpha, categorized as weak (*α* < 0.70), acceptable (*α* = 0.70–0.80), and strong (*α* > 0.80) [42]. Pearson correlation analysis examined the relationship between foot care knowledge and self‐care behavior [43]. Multivariate regression analysis, employing the enter method and excluding cases pairwise, was used to identify key psychosocial domains influencing behavioral intention to use the smart diabetic foot screening system [44].

## 4 Results

### 4.1 Quantitative Results

#### 4.1.1 Participant Characteristics

Of the 125 individuals invited to participate in the study, 80 (64%) enrolled and completed three questionnaires at baseline. The participant cohort had a mean age of 64.18 ± 9.71 years, with the majority being male and diagnosed with type 2 diabetes (Table 2).

**TABLE 2:** Participant characteristics.

	Participants with diabetes *N* = 80
Age, mean (SD), y	64.18 ± 9.71
Women	37 (46.25%)
Type 2 diabetes	80 (100%)
Duration of diabetes, years	
< 10 years	47 (58.75%)
11–20 years	21 (26.25%)
21–30 years	10 (12.5%)
> 30 years	2 (2.5%)
History of foot ulcers	5 (6.25%)
Education	
High school or below	61 (76.25%)
College	17 (21.25%)
Master or above	2 (2.5%)
Foot self‐checking frequency	
Everyday	29 (36.3%)
Once a week	6 (7.5%)
Every month	1 (1.3%)
Never	39 (48.8%)
Others	5 (6.3%)
UTAUT domain, scores	
Performance expectancy	3.83 ± 0.89
Effort expectancy	4.22 ± 0.91
Attitude	3.78 ± 0.79
Social influence	3.69 ± 1.21
Self‐efficacy	3.84 ± 1.06
Anxiety	1.65 ± 0.96
Facilitating conditions	3.83 ± 1.07
Behavioral intention	3.34 ± 1.18

*Note:* Data are presented as mean ± SD or number (%).

### 4.2 Relationship Between Foot Care Knowledge and Self‐Management Behaviors

Our analysis revealed a significant but modest positive correlation between foot care knowledge and adherence to recommended self‐care behaviors (*r* = 0.31 and *p* < 0.01). Despite participants demonstrating high levels of foot care knowledge (mean score = 17.19, SD = 2.59, out of 20; 86.2% correct response rate), their adherence to recommended practices was lower (mean score = 21.74, SD = 7.44, out of 35). Specifically, only 36.3% of participants conducted daily foot self‐examinations, whereas 48.8% reported no regular inspection routines.

### 4.3 UTAUT Analysis

UTAUT analysis revealed significant positive correlations between behavioral intention and psychosocial factors, with attitude showing the strongest correlation (*r* = 0.72 and *p* < 0.01), followed by performance expectancy (*r* = 0.52 and *p* < 0.01), facilitating conditions (*r* = 0.43 and *p* < 0.01), effort expectancy (*r* = 0.42 and *p* < 0.01), self‐efficacy (*r* = 0.41 and *p* < 0.01), and social influence (*r* = 0.23 and *p* < 0.05). Anxiety showed no significant correlation (*r* = −0.14) (Table 3). Multiple regression analysis identified attitude (*β* = 0.66 and *p* < 0.001) and facilitating conditions (*β* = 0.28 and *p* < 0.05) as significant predictors, collectively explaining 57% of the variance in behavioral intention (adjusted *R* ^2^ = 0.57 and *p* < 0.001). In the multiple regression analysis, the standardized coefficients (*β*) indicate the relative importance of each predictor variable in influencing the outcome. The adjusted *R* ^2^ value measures how well the model explains the variation in the outcome variable, with higher values indicating a better model fit. Our analysis revealed that attitude and facilitating conditions are significant predictors of behavioral intention to adopt the technology. The strong positive relationship between attitude and adoption intention (*β* = 0.66) underscores the importance of promoting a favorable perception of the technology among potential users. This could be achieved through targeted educational campaigns or user testimonials that highlight the benefits and ease of use of the technology. Furthermore, the role of facilitating conditions (*β* = 0.28) emphasizes the need for adequate support systems, such as technical assistance and user training, to facilitate successful adoption.

**TABLE 3:** Results from bivariate and multiple regression analyses with behavioral intention.

Psychosocial factor	Bivariate correlation with behavioral intention (*r*)	Multiple regression to predict behavioral intention		
		Standardized regression coefficient (*β*)	Strongest model (*β*)	Model adjusted *R* ^2^
Performance expectancy	0.52**	0.11		
Effort expectancy	0.42**	−0.06		
Attitude	0.72**	0.66	0.66**	0.57**
Social influence	0.23*	−0.08		
Self‐efficacy	0.41**	−0.04		
Anxiety	−0.14	−0.11		
Facilitating conditions	0.43**	0.28	0.28*	

* *p* < 0.05.

** *p* < 0.01.

### 4.4 Qualitative Results

Twenty participants completed semistructured interviews (median duration: 20.5 min and range: 15.5–35.4). Thematic analysis revealed three main themes with nine corresponding subthemes (Table 4): technology acceptance perceptions, implementation support system, and self‐management patterns. In reporting our findings, we differentiated between direct verbatim quotations from individual participants (presented in quotation marks with specific participant identifiers) and investigator summaries of similar sentiments expressed by multiple participants (presented without quotation marks but with notation of relevant participant identifiers). This approach maintained methodological transparency although accurately representing both individual voices and collective patterns across the dataset.

**TABLE 4:** Main themes identified in semistructured interviews.

Main theme	Subtheme 1	Subtheme 2	Subtheme 3
Technology acceptance perceptions	Perceived benefits	Personal beliefs	Previous ulcer experiences
Implementation support system	Resource availability	Technical support	Social support system
Self‐management patterns	Current foot care practices	Self‐management understanding	Knowledge–behavior gap

#### 4.4.1 Main Theme 1: Technology Acceptance Perceptions

##### 4.4.1.1 Subtheme 1.1: Perceived Benefits

Participants identified convenience and efficiency as key advantages of the FootHow system. For example, one participant noted, “It's really just for measurement! This way would be very convenient, no need to wait so long at the hospital…” (P6). Another participant emphasized its precision, stating, “The app reminds me to check my levels and shows me patterns I wouldn't notice myself. Before using it, I thought I was following my care plan, but I was missing a lot” (P12). Several other participants (P8 and P10) also highlighted the convenience and precision of the system, aligning with their positive perceptions of its benefits.

##### 4.4.1.2 Subtheme 1.2: Personal Beliefs

Participants expressed varied perspectives about technology adoption, primarily concerning usability and confidence. One participant noted challenges with the interface, stating, “Need to coordinate with app usage, which might be difficult for elderly people to use. Although it seems simple to operate, there are some interface design issues…” (P12). Similar concerns were shared by another participant (P13), who highlighted the potential difficulties in using the technology. Another participant voiced concerns about operation, stating, “I'm worried! I'm afraid of making mistakes. When measuring data, it's quite scary. I don't dare to use it myself” (P72). This sentiment was echoed by other participants (P75) who expressed anxiety about using the device independently.

##### 4.4.1.3 Subtheme 1.3: Previous Ulcer Experiences

Previous foot complications significantly influenced adoption perspectives. Participants with past ulcers showed proactive interest. For example, one participant noted, “I had ulcers before because of numbness … I don't want this to happen again, so I would consider using this device” (P9). Several other participants (P10 and P12) expressed similar sentiments, highlighting their past experiences as a motivation for considering technology adoption. Others based their adoption decisions on potential future needs. One participant stated, “I don't have any foot problems now, but if I'm hospitalized for foot ulcers one day, I might consider using it” (P7). This perspective was shared by multiple participants (P8, P11, and P14) who similarly considered future health scenarios as a factor in their willingness to adopt the technology.

#### 4.4.2 Main Theme 2: Implementation Support System

##### 4.4.2.1 Subtheme 2.1: Resource Availability

Resource concerns primarily focused on cost and equipment accessibility. Participants expressed financial concerns about the device. For example, one participant noted, “Currently the price might be too high, not sure if I can afford to use it” (P9). Similar financial concerns were shared by other participants (P29) who highlighted the cost as a barrier to adoption. Additionally, participants explored options for shared usage to mitigate costs. One participant asked, “Can multiple people use this device? If we buy one, can the whole family use it?” (P12). This inquiry was echoed by multiple participants (P13 and P73) who considered shared usage as a viable strategy to make the device more accessible.

##### 4.4.2.2 Subtheme 2.2: Technical Support

Technical assistance and training emerged as crucial implementation factors. Participants expressed concerns about device operation. For example, one participant noted, “Will there be any mistakes? Because I'm getting old, I'm worried about operating these devices, so I'm afraid the data might not be recorded or completely lost” (P3). Similar concerns were shared by other participants (P4 and P42) who highlighted the importance of reliable device operation. The need for training support was also emphasized. One participant stated, “If there's technical support and someone to teach us how to use it properly, it would be better” (P6). This sentiment was echoed by multiple participants (P8, P10, and P12) who underscored the importance of having adequate training and technical support for effective device use.

##### 4.4.2.3 Subtheme 2.3: Social Support System

Support from family and healthcare providers played a significant role in influencing adoption decisions. Participants emphasized the importance of professional and family input. For example, one participant noted, “If the doctor or family members want me to use it, I would consider using this device because I might benefit from it.” (P4). Similar sentiments were expressed by other participants (P5 and P7) who highlighted the influence of social support on their willingness to adopt the technology. However, participants also noted potential challenges with support. One participant stated, “When I ask my son to help me measure, he gets impatient” (P72). This sentiment was shared by other participants who faced difficulties in receiving consistent support from family members (P21 and P38).

#### 4.4.3 Main Theme 3: Self‐Management Patterns

##### 4.4.3.1 Subtheme 3.1: Current Foot Care Practices

Participants reported varying levels of engagement in foot care practices. Some incorporated checks into their daily activities. For example, one participant noted, “I don't have a habit of checking. But nowadays when I wash my feet, I will take a look if there are any injuries” (P12). Several other participants (P9 and P15) also reported incorporating foot checks into routine activities. In contrast, others recognized inadequate monitoring of their feet. One participant stated, “I check my feet once every 3 months, but I know I should check more frequently” (P7). This sentiment was shared by multiple participants (P10, P11, and P14) who acknowledged the need for more regular foot inspections.

##### 4.4.3.2 Subtheme 3.2: Self‐Management Understanding

Participants demonstrated a varied understanding of foot care requirements, often citing insufficient guidance. Some noted a lack of instruction regarding the necessity of daily foot checks. For example, one participant stated, “I don't know if I need daily foot checks. The doctor hasn't told me to do so …” (P10). Similar sentiments were expressed by other participants (P16, P60, and P62) who highlighted the absence of clear guidance from healthcare providers. Others reported receiving minimal foot care education. One participant noted, “The doctor never told me I need to check my feet. They focus on controlling diet and diabetes” (P2). This perspective was shared by multiple participants (P3 and P4) who felt that their healthcare providers emphasized other aspects of diabetes management over foot care.

##### 4.4.3.3 Subtheme 3.3: Knowledge–Behavior Gap

Participants acknowledged a disconnection between their awareness of foot care importance and their actual practices. This gap was evident in statements such as “I know exactly what I should be doing—checking my feet, watching what I eat, exercising—but knowing and doing are completely different things. Some days I just can't bring myself to follow through” (P7). Another participant noted, “Even though I know about foot care, I still don't do regular checks” (P8). These sentiments were shared by multiple participants (P10, P12, and P14) who recognized the discrepancy between their knowledge and actions.

## 5 Discussion

This mixed‐methods investigation provides novel insights into smart technology adoption in diabetic foot care management. A significant finding was the discrepancy between knowledge and implementation; despite high levels of foot care knowledge, participants demonstrated limited translation to self‐management behaviors. The modest correlation between knowledge and behavior challenge assumptions about knowledge‐based interventions driving behavioral change. Notably, attitude and facilitating conditions emerged as principal predictors of technology adoption intention, explaining 57% of the variance. Our qualitative themes provide valuable context for understanding the significant predictors identified in our regression analysis. The ‘Technology Acceptance Perceptions' theme sheds light on the attitude factor (*β* = 0.66), which emerged as our strongest quantitative predictor. Participants' discussions about perceived benefits and personal beliefs revealed the underlying attitudinal components that influenced adoption intentions. Similarly, the “Implementation Support System” theme offers insights into the facilitating conditions (*β* = 0.28) that significantly predicted behavioral intention. Participants' emphasis on resource availability and technical support aligns with the quantitative importance of environmental factors in technology adoption decisions. The ‘Self‐management Patterns' theme, particularly the knowledge–behavior gap subtheme, helps explain why certain factors, such as performance expectancy, showed strong bivariate correlations but did not emerge as independent predictors in the multivariate model. This mixed‐methods integration enhances our understanding of the psychological and contextual factors driving technology adoption among patients with diabetes.

Previous research has established various degrees of correlation between foot care knowledge and self‐care behaviors among patients with diabetes. Ko et al. [31] investigated this relationship in 91 patients with diabetes using validated foot care knowledge and behavior scales, reporting a correlation coefficient of 0.33 (*p* < 0.05). Similarly, Lin [45] conducted an interventional study with 102 patients with diabetes divided into experimental and control groups, demonstrating that posteducation knowledge significantly correlated with self‐care behaviors (*r* = 0.25). Our findings revealed a comparable modest correlation between foot care knowledge and behavior (*r* = 0.31 and *p* < 0.05), consistent with these earlier studies despite slight variations in correlation strength. A notable finding was the substantially higher foot care knowledge levels in our study population, with an average correct response rate of 86.2% compared to 67.6% reported by Ko et al. [31] This difference may be attributed to higher educational levels in our study population (57.6% above junior high school vs. 5.5% in Ko's study) and increased general health awareness in contemporary society. However, despite enhanced knowledge levels, the translation to actual foot care behaviors remained suboptimal.

Qualitative findings provided deeper insights into this knowledge–behavior gap. Interviews revealed that 63% of participants considered foot self‐examination unnecessary, with 30% perceiving it as burdensome. Participants expressed low risk perception (“foot ulcers won't happen to me”) and perceived inconvenience of regular foot examination. Notably, 13% of participants questioned the necessity of foot self‐examination, whereas 5% reported never receiving foot care education. These findings align with Paudel et al.'s [46] systematic review, which found that foot care adherence (42%) was notably lower compared to other self‐management behaviors such as blood glucose monitoring (65%), medication adherence (64%), physical activity (53%), and diet management (48%). This consistent pattern of lower prioritization of foot care in diabetes self‐management behavior highlights the need for targeted interventions to improve foot care adherence.

In the present study, the regression analysis revealed that attitude and facilitating conditions were significant predictors of behavioral intention to adopt the FootHow system, collectively explaining 57% of the variance. These findings both align with and diverge from those reported by Macdonald et al. [47] who investigated smart insole adoption among regional adults with diabetes. Specifically, Macdonald et al. found that attitude, self‐efficacy, performance expectancy, and effort expectancy combined explained 51% of the variance in adoption intention. Interestingly, although attitude emerged as a consistent predictor across both studies, our findings highlighted the distinct importance of facilitating conditions rather than self‐efficacy and expectancy factors. This variation in predictive factors may be attributed to the technological differences between the FootHow system, which incorporates imaging capabilities, compared to smart insoles. The emphasis on facilitating conditions in our study suggests that more complex healthcare technologies may require greater consideration of support systems and resource availability for successful implementation [48]. These findings contribute to our understanding of how different smart technologies in diabetic foot care may necessitate varying approaches to support adoption in regional settings.

The implications of this study extend to healthcare policy considerations, particularly regarding the integration of smart technologies into existing diabetes management frameworks. The substantial variance in adoption intention explained by facilitating conditions highlights the critical need for supportive healthcare infrastructure. Policy makers should consider developing comprehensive guidelines that address not only the technical implementation of smart healthcare technologies but also the necessary support systems for sustainable adoption [49, 50]. This study's findings regarding the role of facilitating conditions align with broader healthcare technology implementation research, suggesting that successful integration requires systematic policy support [51]. Moreover, given that only 36.3% of participants in our study reported conducting daily foot examinations despite high knowledge levels, policies should focus on creating enabling environments that support technology adoption. This could include considerations for reimbursement mechanisms [52], technical support infrastructure [53], and healthcare provider training programs [54]. Special attention should be paid to addressing disparities in technology access and support, particularly among older adults and those in regional areas who may face additional barriers to technology adoption [55].

Several limitations warrant consideration. The cross‐sectional design limits examination of causal relationships between knowledge, behavior, and technology adoption intention. Additionally, recruitment from a single regional area may limit generalizability to other contexts. Future research should employ longitudinal designs to evaluate relationships between adoption intention and actual usage behavior. Key areas for investigation include: FootHow implementation across diverse healthcare settings, healthcare providers' role in supporting technology adoption, and integration of smart technologies with existing diabetes education programs to address the knowledge–behavior gap. Cost‐effectiveness analyses across different healthcare contexts would also inform large‐scale implementation decisions.

## 6 Conclusion

This mixed‐methods study reveals crucial insights into technology adoption in diabetic foot care management. The modest correlation between foot care knowledge and self‐management behavior, despite high knowledge levels, underscores limitations of knowledge‐based interventions alone. Attitude and facilitating conditions emerged as key predictors of technology adoption intention, suggesting successful implementation of smart healthcare technologies, requires addressing both personal and environmental factors. Although the FootHow system shows promise for enhancing foot care practices, its effective implementation necessitates consideration of attitudinal barriers and supportive infrastructure. These findings provide practical guidance for healthcare providers and policy makers in implementing smart technologies for diabetes care, emphasizing the need for comprehensive approaches that integrate both individual and systemic factors to improve foot care outcomes.

## Author Contributions

**Ting‐Ting Yeh:** conceptualization, methodology, data curation, formal analysis, writing – original draft, writing – review and editing, project administration, funding acquisition. **Jawl‐Shan Huang:** resources, supervision, validation, writing – review and editing. **Yun‐Chieh Chou:** data curation, formal analysis, writing – original draft.

## Ethics Statement

Ethics approval was obtained from Chang Gung Memorial Hospital Institutional Review Board (IRB: 202300512B0). All participants provided informed consent to participate.

## Conflicts of Interest

The authors declare no conflicts of interest.

## Data Availability

The data that support the findings of this study are available from the corresponding author upon request. The data are not publicly available due to ethical restrictions.
